# Supplementary material for: Comprehensive evaluation of drought stress on medicinal plants: a meta-analysis
Source: PeerJ. 2024 Jul 22;12:e17801. doi: 10.7717/peerj.17801 (PMC11271654; doi:10.7717/peerj.17801)
Supplement: Supplemental Information 3 [file peerj-12-17801-s003.docx]

**Meta-analysis rationale**

1. **The rationale for conducting the systematic review / meta-analysis.**

Drought stress significantly impacts plants by altering their agronomic physiological and biochemical processes, which can drastically reduce the growth and productivity of crops. Many studies have identified key outcomes of drought stress on plants, but there are differing results among studies regarding its effects on certain parameters. For instance, essential oil, chlorophyll and antioxidant contents changes under drought stress have varying results in medicinal plants. The theoretical knowledge suggests that drought conditions are likely to increase the essential oil and antioxidant content in plants, while decreasing chlorophyll levels. Although most studies support these outcomes, there are also studies suggest differently, this indicate that either bias effect or complexity and variability of plant responses to drought stress.

Additionally, there is not enough knowledge about which parameters better respond to drought stress. For example, the enzymatic and non-enzymatic antioxidants are affected by drought may differ but we don’t know their effect sizes. In most studies, non-enzymatic antioxidants in medicinal plants are suggested to act as a defense mechanism against drought, leading to an increase in their synthesis; however, their level of effectiveness has not been compared with enzymatic antioxidants.

Another aspect of this study is to examine 10 different parameters together, comparing how each is affected by drought to assess their interactions. Previous studies have not examined all these parameters simultaneously. For instance, researchers have either focused on enzymatic antioxidants or evaluated non-enzymatic antioxidants. Non-enzymatic antioxidants are commonly studied in medicinal plants, with only a few studies looking into flavonoid, phenol and relatively few studies focusing enzymatic antioxidants contents. Most researchers consider that it is sufficient for medicinal plants to determine non enzymatic antioxidants as a good indicator for drought or other abiotic stress factors. However, this meta-analysis will investigate whether this is true or not, aiming to confirm its accuracy. Conducting a meta-analysis is a highly suitable and reliable method for this type of comparisons.

1. **The contribution that it makes to knowledge in light of previously published related reports, including other meta-analyses and systematic reviews.**

Currently, there is a lack of studies and meta-analyses that particularly examine the effectiveness of different parameters of agronomic, phycological and biochemical in medicinal plants which contains high essential oil and seconder metabolites. The results of our meta-analysis have uncovered the effect sizes of parameters those react better to drought-induced stress. Study results is going to help for future studies about which parameters should be prioritized when evaluating the impact of drought and other abiotic stresses.
